# Seasonal hypoxia and nitrate depletion in water chestnut (*Trapa natans*) beds: Implications for nitrogen cycling

**DOI:** 10.1002/jeq2.70319

**Published:** 2026-11-02

**Authors:** Wade Simmons, Bernd Blossey, Michael Todd Walter

**Affiliations:** ^1^ Department of Natural Resources and the Environment Cornell University Ithaca New York USA; ^2^ Department of Biological and Environmental Engineering Cornell University Ithaca New York USA

## Abstract

Dense beds of floating macrophytes can alter water quality by modifying dissolved oxygen (DO) and nitrogen (N) dynamics. Water chestnut (*Trapa natans* L.), a non‐native annual macrophyte, forms extensive seasonal beds in freshwater systems in North America. The species is intensively managed to reduce negative impacts on native species, yet its effects on DO and nitrate (NO_3_^−^) remain poorly studied. We quantified DO and NO_3_^−^‐N concentrations inside and outside of three riverine *T. natans* beds in New York over three growing seasons. In addition, we assessed denitrification using field‐collected water and sediment in laboratory microcosm incubations. During summer months, DO of water from within *T. natans* beds was 76% lower on average (1.28 mg L^−^^1^) compared to outside beds (5.43 mg L^−^^1^). For NO_3_^−^‐N, concentrations averaged 0.45 (2017) and 0.11 (2018) mg L^−^^1^ outside beds, but were depleted to 0.10 (2017) and 0.01 (2018) mg L^−^^1^ within beds, 77%–88% lower than outside beds. In 2018, 60% of samples, mostly from within beds of *T. natans*, were below the analytical detection limit of NO_3_^−^‐N (0.02 mg L^−^^1^). Laboratory microcosm incubations did not detect consistent N_2_ production, despite experimental nitrate enrichment. Together, these results show that *T. natans* beds create seasonally hypoxic, nitrate‐depleted conditions. However, weak and inconsistent N_2_ production in our incubation experiments indicates that additional studies are needed to further assess the fate of nitrogen within *T. natans* beds and whether management may involve potential tradeoffs between habitat quality and nutrient cycling.

## Plain Language Summary

Water chestnut is an invasive aquatic plant that forms dense floating mats on the surface of freshwater ecosystems. We studied how these plant beds affected oxygen and nitrate levels at three sites in New York over multiple years. During the summer, water chestnut beds were associated with reduced dissolved oxygen and nitrate concentrations, often creating severely hypoxic conditions that can be stressful for aquatic life. The extent of these impacts depended on how water moved and mixed, remaining localized in well‐mixed systems but extending downstream where mixing was limited. Although nitrate concentrations were greatly reduced within water chestnut beds, our experiments did not provide clear evidence that this nitrate was permanently removed through denitrification. These findings show that water chestnut can substantially alter water quality and highlight the need for further research to determine the fate of depleted nitrate in invaded freshwater ecosystems.

**Abbreviation**
MIMS: membrane inlet mass spectroscopy

## 1 INTRODUCTION

Freshwater ecosystems support high levels of biodiversity and provide key ecosystem services, yet are among the most degraded environments on Earth (Carpenter et al., 2011; Darwall et al., 2018; Dudgeon & Strayer, 2025; McAllister et al., 1997). Among the multiple stressors affecting these systems, some non‐native species are especially important because they act as ecosystem engineers, altering physical and chemical conditions in ways that restructure biological interactions and ecosystem properties and can shift systems toward alternative stable states (Emery‐Butcher et al., 2020; Jones et al., 1994; Matsuzaki et al., 2009; Scheffer et al., 2003; Strayer, 2009, 2010). Introduced aquatic plant species, in particular, can present acute and complex management and conservation challenges because their large biomass can simultaneously interfere with human uses of waterways, provide habitat for organisms, and function as “hot spots” of ecosystem metabolism and nutrient transformation (Caraco et al., 2006; Dudgeon & Strayer, 2025; Rascio, 2002).

Regulation of oxygen (O_2_) by aquatic plants is a key mechanism that can structure aquatic environments, influencing habitat quality and biogeochemical cycling. Dissolved O_2_ concentrations in aquatic environments are much lower than atmospheric concentrations due to the physical and chemical relationships between water and dissolved gases, making biota in aquatic systems particularly vulnerable to changes in O_2_ dynamics (Caraco et al., 2006). As primary producers, aquatic plants generate O_2_ as a byproduct of photosynthesis, but contributions to the dissolved oxygen (DO) budget can be positive (Rejmánková et al., 2018) or negative (Caraco & Cole, 2002) depending on plant growth form and functional traits (Caraco & Cole, 2002; Caraco et al., 2006). Submerged plants can oxygenate waters directly via photosynthesis, but plants with floating leaves can vent O_2_ directly back to the atmosphere, limiting their contribution to aquatic DO budgets (Caraco et al., 2006). Dense surface cover by floating leaves can also reduce light availability (Perna & Burrows, 2005; Villamagna & Murphy, 2010), limiting photosynthesis by submerged plants and phytoplankton, while respiration by plants and microbial communities can increase oxygen demand, further reducing DO concentrations.

Aquatic DO concentrations can be strongly affected by both a change in the dominant growth form of aquatic plant communities, as well as by the physiological capacity of plants with floating parts to translocate O_2_ to tissues below the water surface. Water lilies, for example, have intricate mechanisms for subsidizing underwater parts with atmospheric O_2_ (Dacey, 1980), but other plants, such as duckweeds, are more limited in their translocation capacity, and use DO to meet respirational demands of underwater structures (Caraco & Cole, 2002; Pokorný & Rejmánková, 1983). At high densities, O_2_ consumption by plants with limited O_2_ transfer abilities can generate hypoxic conditions, with cascading consequences for habitat quality for aquatic biota. When other stressors, such as nutrient loading (Coetzee & Hill, 2012; Portielje & Roijackers, 1995), contribute to the development of large, dense populations of floating macrophytes with limited O_2_ translocation systems, these conditions are considered a key threat to aquatic ecosystems (Scheffer et al., 2003).

By influencing O_2_ concentrations, aquatic macrophytes may have an important influence on nitrogen (N), which is both an essential nutrient and a contributor to eutrophication and declines in water quality (Diaz & Rosenberg, 2008). Reductions in DO can promote denitrification via microbially mediated reduction of inorganic N to gaseous forms. Denitrification occurs in low O_2_ conditions and is largely limited by available NO_3_^−^ and labile carbon substrates. In aquatic environments, this process commonly occurs in benthic sediments, but can also occur at anoxic microsites of other substrates, including suspended sediments, algal mats, and on surfaces of vascular plants (Reisinger et al., 2016; Schaller et al., 2004). When DO is extremely low, NO_3_^−^ is often reduced fully to the inert gas, dinitrogen (N_2_) (Groffman et al., 2006). Unlike assimilatory pathways of N loss, which represent temporary storage in organic bonds, denitrification represents a permanent loss of N to the atmosphere and can play a key ecosystem service by mitigating excess N.

In northeastern North America, the non‐native aquatic plant water chestnut, *Trapa natans* L. (Lythraceae), is an example of an ecosystem engineer capable of strongly influencing DO in freshwater habitats (Caraco & Cole, 2002; Caraco et al., 2006; Tall et al., 2011). This highly productive annual plant roots in sediments and produces multiple floating rosettes that seasonally transform open‐water areas into floating beds of dense vegetation. During peak biomass, *T*. *natans* can block >95% of incoming light (Hummel & Kiviat, 2004) and strongly limit recreational water access. Adventitious roots along stems in the water column have large respirational O_2_ demands that the plant is physiologically unable to meet with internal O_2_ translocation, with potentially large impacts on DO concentrations. These functional traits of *T. natans* in concert with physical changes to aquatic habitats can create strongly hypoxic conditions with DO <2 mg L^−1^, which can be lethal to many aquatic organisms (Caraco & Cole, 2002; Goodwin et al., 2008).

The ecosystem engineering capabilities of *T. natans* have been linked to N cycling changes in the freshwater tidal portion of the Hudson River of New York, where depletions of inorganic water column N and extremely high denitrification rates have been recorded (Tall et al., 2011). Despite occupying only a small percentage of the river and an active growing season of only a few months, *T. natans* beds are estimated to account for ∼25% of the annual N removal of the entire Hudson River (Lampman et al., 1999). However, tidal and nontidal freshwater systems differ fundamentally in hydrologic exchange and nutrient delivery. In the Hudson River, tides replenish DO and inorganic nitrogen twice daily, maintaining a regular supply of reactants that may enhance biogeochemical processing within *T. natans* beds. In contrast, nontidal systems, where the vast majority of *T. natans* invasions take place, lack this periodic resupply, raising the possibility that oxygen depletion, nitrate depletion, and denitrification differ substantially in magnitude and spatial extent.

Understanding whether and how oxygen and nitrogen dynamics differ between tidal and nontidal systems is important for both ecological understanding and invasive species management. For example, reducing abundance and coverage of *T. natans* beds will likely alleviate hypoxic conditions, but may simultaneously reduce N removal. To better characterize impacts of *T. natans* on the aquatic environment, we monitored DO and NO_3_^−^‐N concentrations in three *T. natans*‐dominated freshwater habitats over three growing seasons in upstate New York, USA. To test whether patterns observed in tidal freshwater systems extend to nontidal habitats, we predicted that (1) DO and NO_3_^−^ concentrations would be lower inside *T. natans* beds relative to nearby uninvaded areas, (2) DO levels would decline to hypoxic levels as *T. natans* surface cover expands but recover as plants senesce at the end of the growing season, and (3) denitrification potential would be greater within *T. natans* beds compared to uninvaded sites.

## 2 METHODS

To assess the influence of *T. natans* on nitrogen and oxygen dynamics, we monitored three large beds (>2 ha) in lotic freshwater sites (Figure 1) during the 2017, 2018, and 2019 growing seasons. We selected sites based on accessibility and for representativeness of *T. natans* beds in the region that are typically found in shallow, slow‐moving habitats. Water depths at sampling locations within beds of *T. natans* ranged from approximately 0.2 to 4.0 m, although many samples were collected in water <1 m deep. Two sites (Lions Park and Fishing Access) occur along the Mohawk River, near Schenectady, New York, close to the first North American point of introduction of *T. natans* (Hummel & Kiviat, 2004). The third site, Red Creek, is located near Lake Ontario, approximately 200 km west of the Mohawk River sites.

**FIGURE 1:**
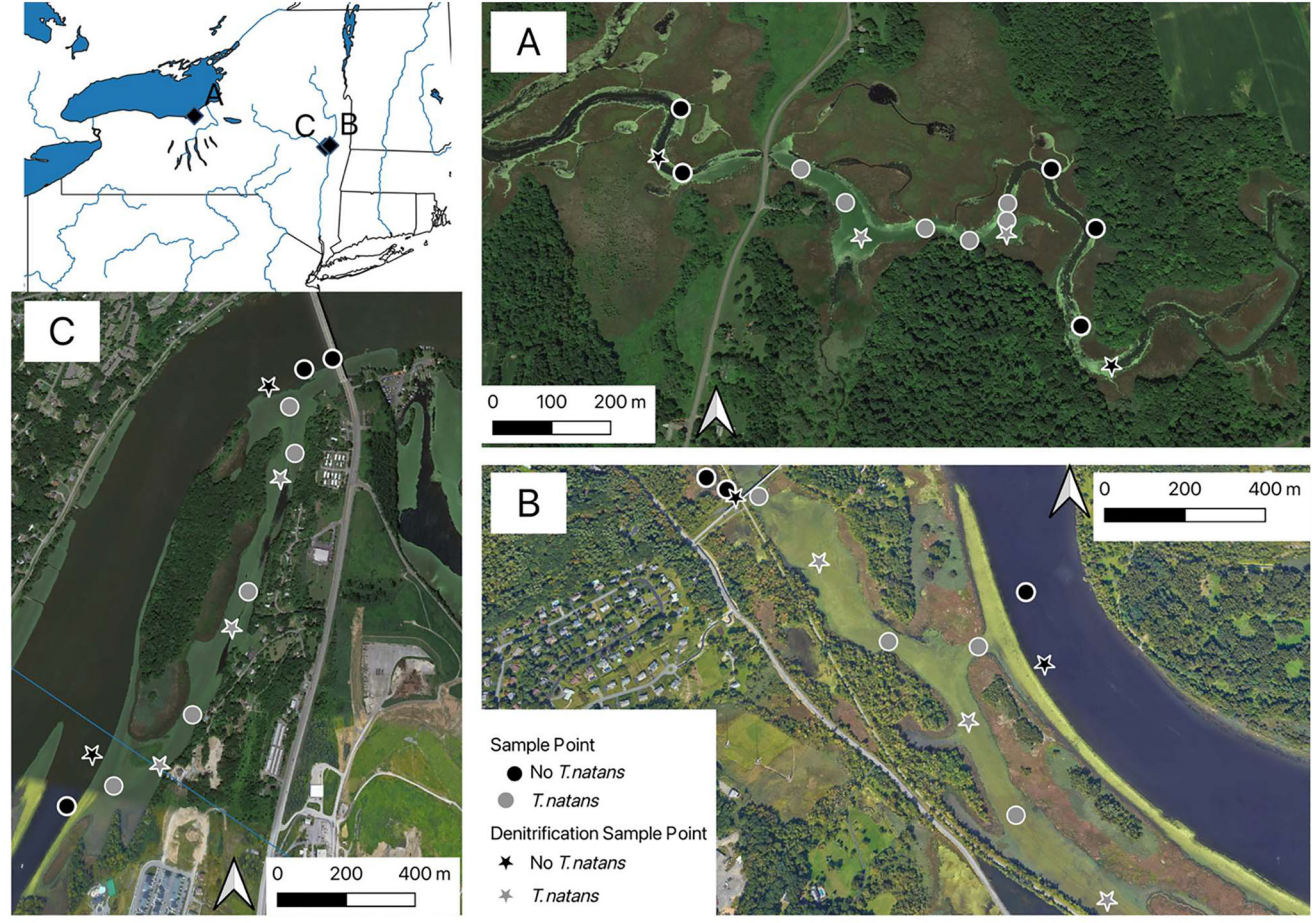
Location of field sites in New York State. (A) Red Creek, (B) Lions Park, and (C) Fishing Access. Satellite imagery from July 2017 shows extent of large beds of *Trapa natans*. Sampling locations are colored according to sampling category, with black circles representing samples outside beds of *T. natans* and gray circles from sample points within *T. natans* beds. Stars represent sample locations for denitrification experiment, which were also used for water analysis.

At Lions Park, *T. natans* occurs in a shallow and wide backwater embayment (≈35 ha) that is fed upstream by Lisha Brook and flows out into the Mohawk River. At Fishing Access, located 3.2 km river downstream of Lions Park, *T. natans* occupies a shallow side channel (≈12.5 ha) that flows around a small island before rejoining the main river. At both Mohawk River sites, water exiting the *T. natans* beds rapidly mixes with the substantially larger river channel. At Red Creek, the *T. natans* bed (≈2.5 ha) has existed for less than a decade compared to decades of occupation at the Mohawk River sites. Red Creek is a short, low gradient waterbody that widens through a cattail (*Typha* spp.) marsh before emptying into Lake Ontario. Unlike at the Mohawk River sites, water exiting the *T. natans* bed does not strongly mix with other waters until it reaches Lake Ontario, thus retaining potential biogeochemical signals generated during passage through the *T. natans* bed.

At each site, we sampled locations upstream of, within, and downstream of *T. natans* beds. Each site had a unique sampling design based on physical characteristics (Figure 1), and locations within the *T. natans* beds are over‐represented relative to upstream and downstream locations. We used a canoe and GeoExplorer 6000 (Trimble, Waypoint Technology) to relocate sampling locations, and visited sites in the 2017, 2018, and 2019 growing seasons. We categorized field visits according to three seasonal development stages of *T. natans*: early (May–June; rosettes emerge on the water surface); mid (July–early September; peak biomass and surface coverage of *T. natans*); and late (October; *T. natans* is senescing). At each sampling location, we recorded DO 10 cm below the surface using a YSI Pro Plus probe (YSI).

In 2017 and 2018, we collected water samples in 125‐mL bottles, which we stored on ice in a dark cooler. Upon returning to the lab, we filtered samples (GF/F Whatman Filters), acidified to pH < 2 with H_2_SO_4_, and stored samples in a 5°C walk‐in cooler (Nor‐Lake Inc.) within 24 h of collection. We analyzed water samples for NO_2_^−^ + NO_3_^−^‐N concentrations using colorimetric methods. In 2017, we used a Lachat Quickchem 8100 flow‐injection analyzer (MDL: 0.01 mg L^−^^1^) (Lachat Instruments), and in 2018, we used a microplate analyzer (MDL: 0.02 mg L^−^^1^) (Doane & Horwáth, 2003). For the 2018 samples, we assigned values below detection to 0.01 mg L^−^^1^.

### 2.1 Denitrification incubations

To explore denitrification dynamics, we selected one point upstream, downstream, and at least two within each *T. natans* bed at each site (Figure 1). We collected 2 L of water 10 cm below the surface and capped bottles under water without bubbles to minimize atmospheric exposure. We collected sediment samples using a petite ponar grab sampler (sample area: 15.24 cm W × 15.24 cm L; volume: 2.4 L; Pine Environmental Services LLC), homogenized samples, and transferred them to a plastic bag. At each sample location, we collected three replicate water and sediment samples and stored them in a dark cooler without ice to maintain temperature and preserve gas chemistry of water samples.

Upon returning to the lab, we assembled incubation chambers in sterile 50‐mL centrifuge tubes (*n* = 15/site) with three replicates each for five incubation times (0, 1, 2, 4, and 8 h) following protocols outlined by Reisinger et al. (2016). We re‐homogenized site sediment and transferred 10 mL to each corresponding incubation tube. After all incubation tubes received sediment, we quickly added 40 mL of gently stirred water from the same site using a syringe modified with tubing to minimize sediment suspension. To minimize atmospheric oxygen contamination for water samples, we capped centrifuge tubes underwater.

We incubated at 20°C, corresponding to the average temperature at field sites at time of sample collection, and kept microcosms in the dark until destructive sampling by unscrewing the cap and drawing ∼36 mL water from near the sediment layer with a syringe modified with tubing. We then quickly transferred all water from the syringe into the bottom of a 12‐mL Exetainer vial (Labco Ltd.), lifting the syringe tubing within the exetainer as it overfilled, ending up with a convex meniscus at the opening. We added 0.2 mL of 50% ZnCl_2_ to each sample to preserve gas chemistry, capped each sample, and then stored samples inverted and submerged underwater in a dark refrigerator until time of analysis. We analyzed samples for dissolved concentrations of N_2_‐N, O_2_, and argon (Ar) using a membrane inlet mass spectrometer (MIMS; Bay Instruments) (Kana et al., 1994). We calculated dissolved N_2_ and O_2_ concentrations from the N_2_‐N:Ar and O_2_:Ar ratios measured by the MIMS by multiplying each gas ratio by the equilibrium concentration of dissolved Ar, calculated from laboratory temperatures and barometric pressure following Hamme and Emerson (2004) and Reisinger et al. (2016). We ran all samples at a standard temperature of 20°C. To assess the role of NO_3_^−^ limitation in denitrification dynamics, we repeated the denitrification incubations for the Mohawk River field sites (Figure 1) in 2019, but enriched NO_3_^−^ concentration of water samples by 5 mg L^−1^ with dissolved KNO_3_^−^. We did not sample Red Creek due to ongoing chemical management of the *T. natans* bed.

### 2.2 Statistical analyses

To evaluate the effects of *T. natans* across study sites, we analyzed DO and NO_3_^−^‐N concentrations separately using linear mixed‐effects models in R (R Core Team, 2025) with the lme4 package (Kuznetsova et al., 2017). For this general analysis, we combined upstream and downstream samples from the two Mohawk River sites into a “No *T. natans*” category because water exiting *T. natans* beds rapidly mixed with the much larger main river channel, preventing upstream and downstream locations from retaining distinct hydrologic conditions created by *T. natans*. Downstream samples from Red Creek were excluded from the overall analysis because they represented a different hydrologic context, where water that had passed through *T. natans* beds experienced minimal dilution prior to downstream sampling. These downstream locations were instead evaluated in separate site‐specific analyses.

For DO, we square‐root transformed the response variable and fit a global model including an interaction between *T. natans* presence (within vs. outside beds) and growing season stage (early, mid, and late), with site and year as fixed effects and sampling location as a random intercept to account for repeated measures. We reduced models through sequential backward elimination, testing nested models with likelihood ratio tests until no nonsignificant terms remained. For this and all subsequent models, we evaluated assumptions of residual normality and homogeneity of variance by visual inspection of diagnostic plots. We then calculated estimated marginal means (EMMs) and pairwise contrasts using the emmeans package (Lenth, 2025).

For NO_3_^−^‐N, we analyzed mid‐growing season separately for 2017 and 2018 due to differences in analytical methods between years. In each year, we fit linear mixed models with log‐transformed NO_3_^−^‐N as the response, *T. natans* presence and site as fixed effects, and sampling location as a random intercept, and reduced models and calculated EMMs as described above.

To evaluate how hydrologic setting (Mohawk River vs. Red Creek) influenced general patterns, we conducted site‐specific analyses examining how the effects of *T. natans* differed among study sites. This two‐tiered approach allowed us to estimate the overall effects of *T. natans* across sites before evaluating spatial patterns unique to individual systems. We analyzed mid‐season DO (all years) and NO^3−^‐N (2017) using three site‐specific comparisons: (1) the two *T. natans* beds in the Mohawk River (Lions Park, Fishing Access) relative to the adjacent main river channel, (2) the upstream tributary and *T. natans* bed at Lions Park, and (3) upstream, *T. natans* bed, and downstream locations at Red Creek. We square‐root transformed DO and log transformed NO^3−^‐N to improve residual normality and stabilize variance. For the Mohawk River comparisons, we fit linear mixed‐effects models for both response variables, with *T. natans* presence as a fixed effect and sampling location as a random intercept. For the Lions Park comparison, inclusion of random intercepts for sampling location resulted in a singular fit; therefore, we fit the final models as linear models with presence of *T. natans* as a predictor. For Red Creek, we fit the final DO model as a linear model because inclusion of a random intercept for sampling location resulted in a singular fit. The NO^3−^‐N model was fit as a linear mixed‐effects model with habitat category (upstream, *T. natans*, downstream) as a fixed effect and sampling location as a random intercept. We evaluated fixed effects in mixed‐effects models using Type II F‐tests with Kenward–Roger approximations for denominator degrees of freedom and evaluated linear models using standard *F*‐tests. We calculated EMMs and conducted pairwise comparisons using the emmeans package and applied Tukey adjustments where we performed multiple pairwise comparisons.

We calculated denitrification rates separately for each sampling location. At each incubation time, we averaged dissolved N_2_ concentrations across three replicate vials and regressed the mean N_2_ concentration against incubation time (h). We calculated dissolved N_2_ concentrations from measured N_2_:Ar ratios following Kana et al. (1994) and Reisinger et al. (2016). We used the slope of each regression as the net rate of N_2_ accumulation (µmol N L^−^^1^ h^−^^1^). We then modeled location‐level N_2_ accumulation as a function of *T. natans* presence and nitrate‐addition, with site included as a random effect.

## 3 RESULTS

For DO, concentrations were affected by growing season stage (*F*_2,187.0 _= 117.68, *p* < 0.001), *T. natans* presence (*F*_1,98.6_ = 54.37, *p* < 0.001), and their interaction (*F*_2,189.4_ = 16.41, *p* < 0.001). DO was lower within *T. natans* beds at all growing season stages relative to uninvaded areas (Figure 2), but differences were only significant during the mid‐growing season when beds became hypoxic. In the summer months, DO averaged 5.43 mg L^−^^1^ (confidence interval [CI]: 4.50–6.46) outside beds and 1.28 mg L^−^^1^ (CI: 0.92–1.71) within beds, a difference of 4.22 mg L^−^^1^ (SE = 0.49, *p* < 0.001). DO declined from early to mid‐season and then partially recovered in both habitats (Figure 2). There were no significant differences between habitats in early season (No *T. natans*: 8.90 mg L^−^^1^, CI: 6.56–11.61; *T. natans*: 7.39 mg L^−^^1^, CI: 5.93–9.01) or late season (No *T. natans*: 6.95 mg L^−^^1^, CI: 5.15–9.03; *T. natans*: 5.90 mg L^−^^1^, CI: 4.75–7.17).

**FIGURE 2:**
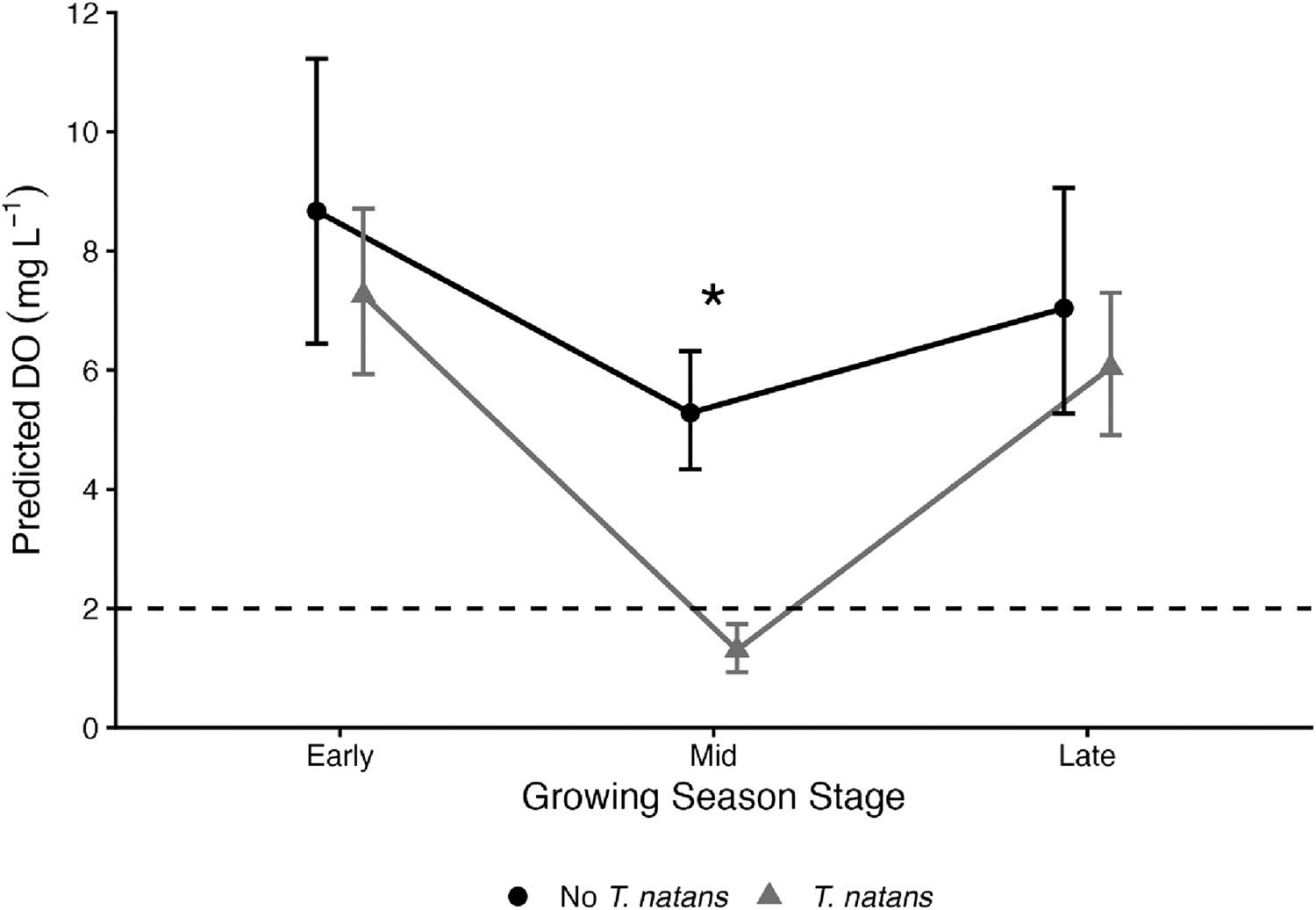
Dissolved oxygen concentrations (mg L^−^^1^) inside (gray triangles) and outside (black circles) *T. natans* beds across growing season stages. Points represent model‐predicted estimated marginal means (±95% confidence interval [CI]) from linear mixed‐effects models (back‐transformed). The dashed line at dissolved oxygen (DO) = 2 mg L^−^^1^ indicates a commonly used hypoxia threshold. An asterisk (*) indicates a significant difference between *T. natans* and No *T. natans* conditions during mid‐season.

Nitrate (NO_3_^−^‐N) concentrations in 2017 differed among sites (*F*_2,26.6_ = 9.29, *p* < 0.001) and were significantly lower within *T. natans* beds (*F*_1,40.3_ = 37.27, *p* < 0.001) (Figure 3). Mean NO_3_^−^‐N concentrations were 0.45 mg L^−^^1^ (CI: 0.30–0.67) in uninvaded areas and 0.10 mg L^−^^1^ (CI: 0.07–0.14) within *T. natans* beds. NO_3_^−^‐N concentrations within *T. natans* beds were 77% lower than in uninvaded areas (SE = 1.08, *p* < 0.001). Site‐level differences were also evident, with NO_3_^−^‐N concentrations significantly higher at Red Creek than at Fishing Access and Lions Park (*p* < 0.01), whereas no differences were detected between Fishing Access and Lions Park (*p* = 0.93).

**FIGURE 3:**
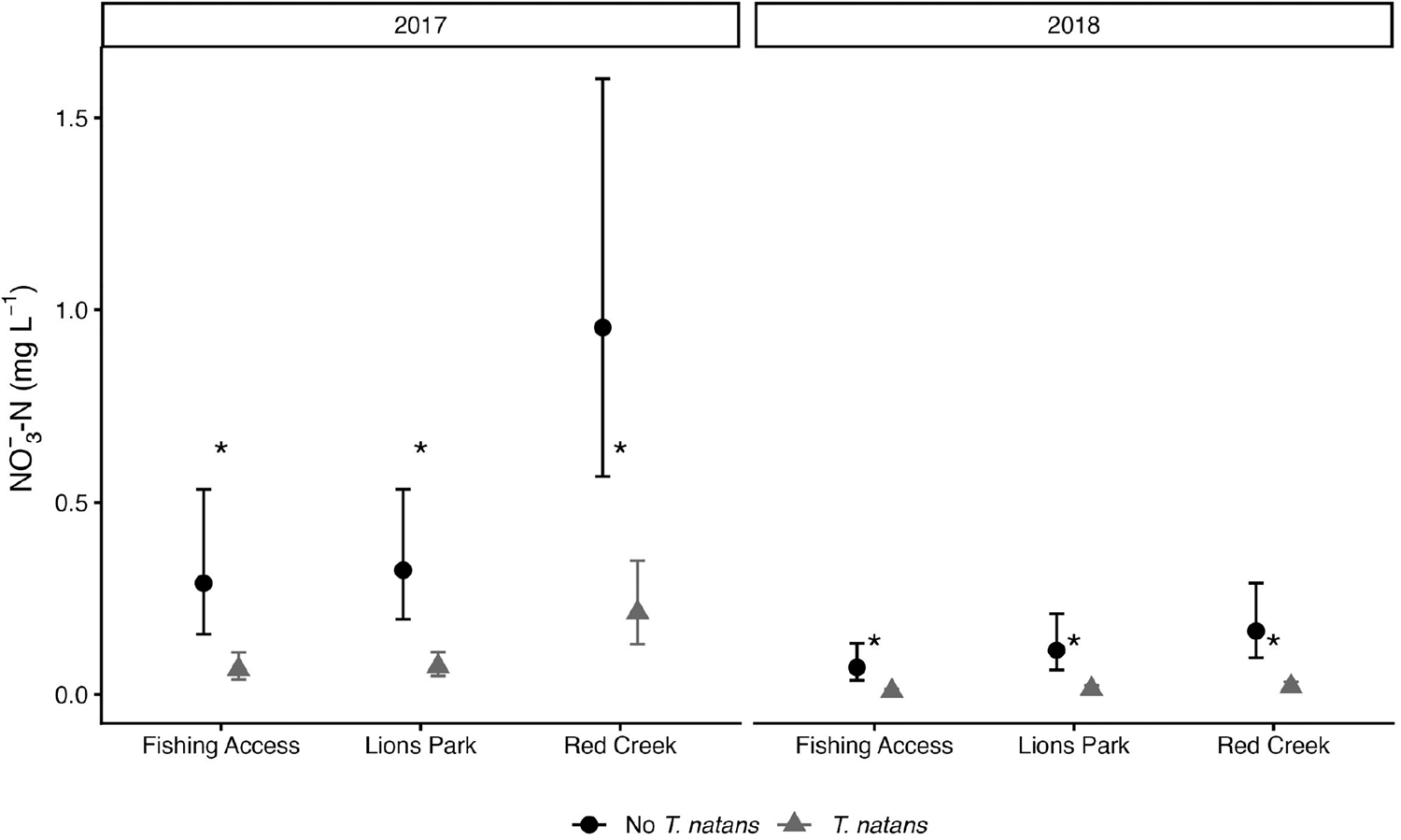
Nitrate (NO_3_^−^‐N, mg L^−^^1^) concentrations in 2017 and 2018 in mid‐season within *T.natans* beds (gray triangles) and uninvaded areas (black circles). Points represent model‐predicted estimated marginal means ±95% confidence intervals obtained from a linear mixed‐effects model fit on the log scale and back‐transformed to the response scale. Confidence intervals for *T. natans* beds are present but appear small in 2018 due to low variance and clustering of values near the analytical detection limit. Asterisk (*) indicates significant differences in NO^3−^‐N concentrations between areas with and without *T. natans*.

Nitrate (NO_3_^−^‐N) concentrations in 2018 again differed among sites (*F*_2,25.2_ = 3.67, *p* = 0.04) and were significantly lower within *T. natans* beds (*F*_1,25.5_ = 57.95, *p* < 0.001) (Figure 3). Mean NO_3_^−^‐N concentrations were 0.11 mg L^−^^1^ (CI: 0.07–0.18) in uninvaded areas and 0.01 mg L^−^^1^ (CI: 0.01–0.02) within *T. natans* beds. Across sites, NO_3_^−^‐N concentrations within *T. natans* beds were 88% lower than in uninvaded areas (SE = 2.26, *p* < 0.001). We observed site‐level differences with NO_3_^−^‐N concentrations higher at Red Creek than at Fishing Access (*p* = 0.03), but detected no differences between other site pairs (*p* = 0.30). Of the 58 mid‐growing season samples collected in 2018, 35 (60%) were below the 0.02 mg L^−^^1^ detection limit, with low values occurring disproportionately within *T. natans* beds (31 of 39 samples, 79%) relative to non‐invaded habitats (four of 19 samples, 21%). Despite the high frequency of low values, nitrate depletion within *T. natans* beds remained consistent with patterns observed in 2017.

Site‐specific analyses supported the general patterns identified in the combined analyses while revealing important differences among hydrologic settings. At Lions Park, DO was 6.34 mg L^−^^1^ lower within *T. natans* beds than in the upstream tributary (*F*_1_,_91_ = 53.98, *p* < 0.001), representing a 76% difference (Figure 4). Nitrate showed a similar pattern, declining by 0.22 mg L^−^^1^ within beds from upstream values (*F*_1_,_25_ = 20.41, *p* < 0.001), also corresponding to a 76% decline. In the Mohawk River, DO was 3.66 mg L^−^^1^ lower within *T. natans* beds than in the adjacent main river channel (*F*_1,88.3 _= 27.08, *p* < 0.001), a 72% decline (Figure 4). Nitrate concentrations were similarly lowered within *T. natans* beds, declining by 0.44 mg L^−^^1^ (89%; F_1,22.3_ = 42.52, *p* < 0.001). At Red Creek, DO differed among sampling locations, with upstream concentrations exceeding both *T. natans* beds (*p* = 0.0034) and downstream locations (*p* = 0.0115). DO declined from 3.86 mg L^−^^1^ upstream to 1.82 mg L^−^^1^ within *T. natans* beds and remained similarly low downstream (1.58 mg L^−^^1^; *T. natans* vs. downstream, *p* = 0.91). Nitrate showed a parallel pattern (*F*_2,14.9 _= 7.19, *p* < 0.01), declining from 0.84 mg L^−^^1^ upstream to 0.23 mg L^−^^1^ within *T. natans* beds and remaining depleted downstream (0.13 mg L^−^^1^). Upstream NO_3_^−^‐N concentrations exceeded both *T. natans* beds (*p* = 0.02) and downstream sites (*p* = 0.01), whereas concentrations within beds and downstream did not differ (*p* = 0.53) (Figure 4).

**FIGURE 4:**
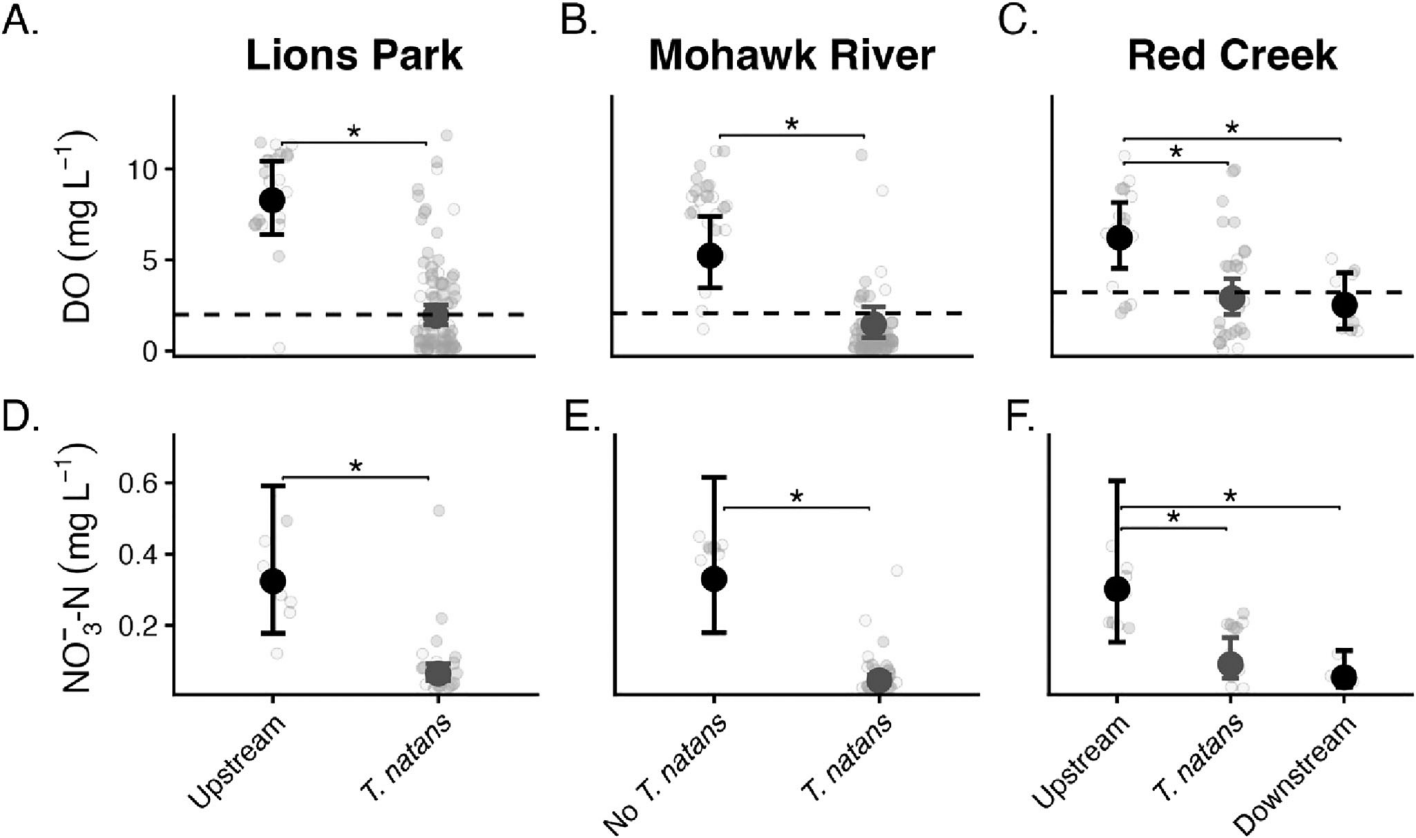
Dissolved oxygen (DO; A–C) and nitrate (NO_3_^−^‐N; D–F) concentrations during the mid‐growing season at *T*. *natans* field sites in New York. Each panel highlights a different hydrologic comparison. At Lions Park (A and D), *T. natans* beds are compared with water entering the site from an upstream tributary. In the Mohawk River (B and E), *T. natans* beds at Fishing Access and Lions Park are compared with adjacent mainstem sampling locations. Red Creek (C and F) was the only site where upstream, *T. natans* bed, and downstream conditions could be compared without substantial dilution from a larger water body. Points represent estimated marginal means (±95% confidence intervals) from the fitted statistical models, and raw data are shown as semitransparent points. Gray symbols indicate samples collected within *T. natans* beds; black symbols represent adjacent locations without *T. natans*. The dashed horizontal line in DO panels denotes 2 mg L^−^^1^, a commonly used hypoxia threshold for aquatic organisms. Asterisk (*) indicates significant pairwise differences between categories connected by brackets.

DO concentrations in denitrification incubations closely matched field conditions, indicating that samples from *T. natans* beds remained hypoxic during experimentation. Rates of N_2_ change during incubations did not differ between invaded and uninvaded samples (*F*_1_,_17_._8_ = 0.19, *p* = 0.67) or between fertilized (2019) and unfertilized (2017) NO_3_^−^ treatments (*F*_1_,_18_._8_ = 0.96, *p* = 0.34; Figure 5). In 2017, unfertilized incubations showed variable N_2_ dynamics, with rates ranging from −6.04 to 4.78 µmol N L^−^^1^ h^−^^1^, and only two of 13 incubations exhibiting rates significantly different from zero. In 2019, fertilized incubations more consistently showed N_2_ accumulation (9 of 10 sites), although rates remained low (range: −0.92 to 2.68 µmol N L^−^^1^ h^−^^1^) and only one site exhibited a significant change over time.

**FIGURE 5:**
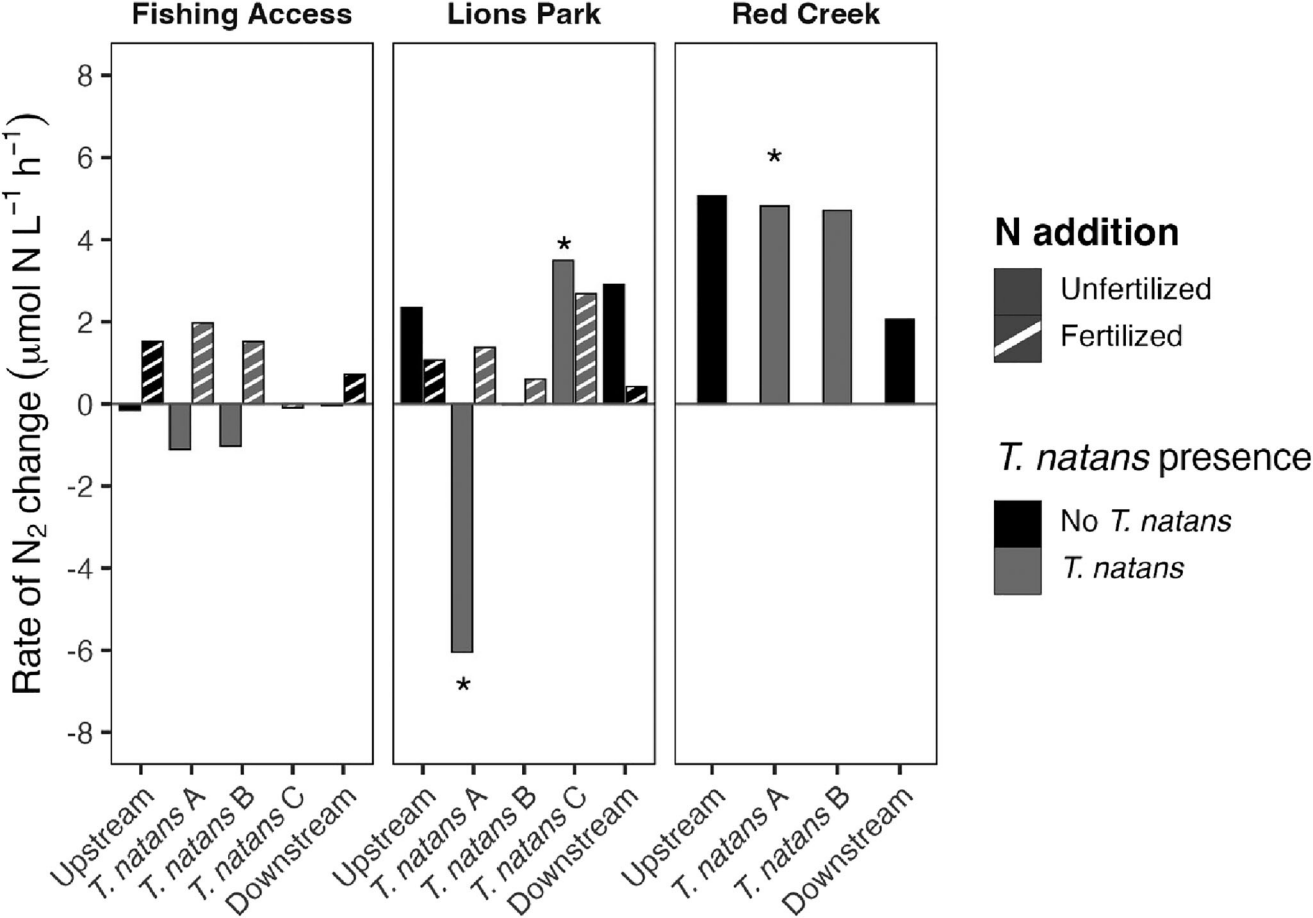
Rates of change in dissolved N_2_ concentrations (µmol N L ^−1^h ^−1^) during 8 h incubations of sediment samples and water collected 10 cm below the water surface in late August (2017) and early September (2019) at sampling points upstream and downstream (“No *T. natans*,” black bars), and within beds of *T. natans* (gray bars; see Figure 1) at three field sites. Nitrate‐enriched treatments (striped bars) received 5 mg L^−^^1^ NO_3_^−^‐N. Asterisk (*) indicates sampling locations where dissolved N_2_ concentrations changed significantly over the incubation period.

## 4 DISCUSSION

Extensive *T. natans* beds not only transform physical habitat features but also generate seasonal water chemistry conditions distinct from uninvaded areas. Across all study sites, we document substantial declines in DO and NO_3_^−^‐N concentrations associated with *T. natans*. Hypoxic and NO_3_^−^‐N‐depleted conditions develop by the summer months and then partially recover with plant senescence in the fall, but not immediately to spring levels. Our data indicate that previously reported ecosystem engineering by *T. natans* from the tidal Hudson River also occurs in inland nontidal waters. Unlike *T. natans* beds in tidal freshwater systems, where regular water exchange mitigates the duration of oxygen depletion, our nontidal study sites experienced prolonged periods of severe hypoxia.

The spatial extent of hypoxic and nitrate‐depleted conditions depended strongly on hydrologic connectivity. At our Mohawk River sites (Fishing Access, Lions Park), declines in DO and NO_3_^−^‐N were largely confined to *T. natans* beds and were not evident at downstream sampling locations. This suggests that these impacts attenuate quickly when mixed with the much larger volume of the main river channel. In contrast, at Red Creek, where water leaving *T. natans* beds remained within the same channel and experienced little dilution until reaching Lake Ontario, water chemistry changes caused by *T. natans* persisted downstream. These downstream effects occurred despite the substantially smaller *T. natans* bed at Red Creek (2.5 ha) relative to the Mohawk River sites (12.5 and 35 ha). At Red Creek, upstream DO was also relatively low, likely because the dense floating rosettes of *T. natans* reduced water movement and promoted the accumulation of duckweed (*Lemna* spp.) and floating organic material. Together, these findings suggest that the ecological consequences of *T. natans* invasions are shaped by interactions between invasion characteristics, such as patch size and plant density (Hummel & Findlay, 2006), and local hydrological conditions (Harvey et al., 2019; Powers et al., 2012).

The loss of nitrate within beds of *T. natans* prompts questions about its fate. We predicted that hypoxic environments associated with *T. natans* would be lower in NO_3_^−^ concentrations and have greater denitrification activity relative to adjacent uninvaded locations. Despite chemical conditions generally considered favorable for denitrification (Groffman et al., 2006), our experimental results did not detect corresponding increases in N_2_ production from sediments and overlying water collected within *T. natans* beds. This discrepancy suggests either that nitrogen was retained through alternative pathways such as plant uptake or microbial immobilization, or that our incubations failed to capture the locations or conditions under which denitrification was occurring. Rates of N_2_ accumulation observed in our incubations (0.64–4.78 µmol N L^−^^1^ h^−^^1^) were substantially lower than those reported for *T. natans* beds in the tidal Hudson River (mean: 7 µmol N L^−^^1^ h^−^^1^; range 0.2–24; Tall et al., 2011). Rather than indicating an absence of denitrification, this difference may reflect fundamental differences in hydrologic setting, methodological approach, or the specific components of the nitrogen cycle captured by the two studies.

One possible explanation is that denitrification remained limited despite hypoxic conditions because another required resource was missing. In addition to low DO conditions, denitrification requires a supply of NO_3_^−^, a carbon substrate, and a microbial community that includes denitrifying functional groups. Suspecting the low concentrations of NO_3_^−^‐N found in our samples might limit denitrification, we investigated NO_3_^−^‐N limitation in our fertilized denitrification incubations of 2019, and found that supplemental NO_3_^−^‐N did not result in increased rates of N_2_ production. While we did not monitor carbon characteristics of site water, measurements in the Hudson River document dissolved organic carbon loads are unaffected by *T. natans* (Hummel & Findlay, 2006), and we do not suspect carbon limitation in our study.

Another possibility is that our incubation design failed to capture the primary locations where denitrification was occurring. A potential source of denitrification activity that was unaccounted for in our experimental design are the epiphytic biofilms that occur on *T. natans* (Figure 6), which are implicated in N cycling for other macrophytes (Castaldelli et al., 2015; Schaller et al., 2004). We did not include live plant materials in our incubation experiments, but epiphytic microbial communities on *T. natans* leaves and roots include multiple bacterial groups with genes known to be involved in production of enzymes important for denitrification (Han et al., 2018). While we currently lack understanding of the ecological function of these microbes, we hypothesize that epiphytic biofilms represent a key, previously unaccounted pathway for nitrogen transformation in *T. natans* beds.

**FIGURE 6:**
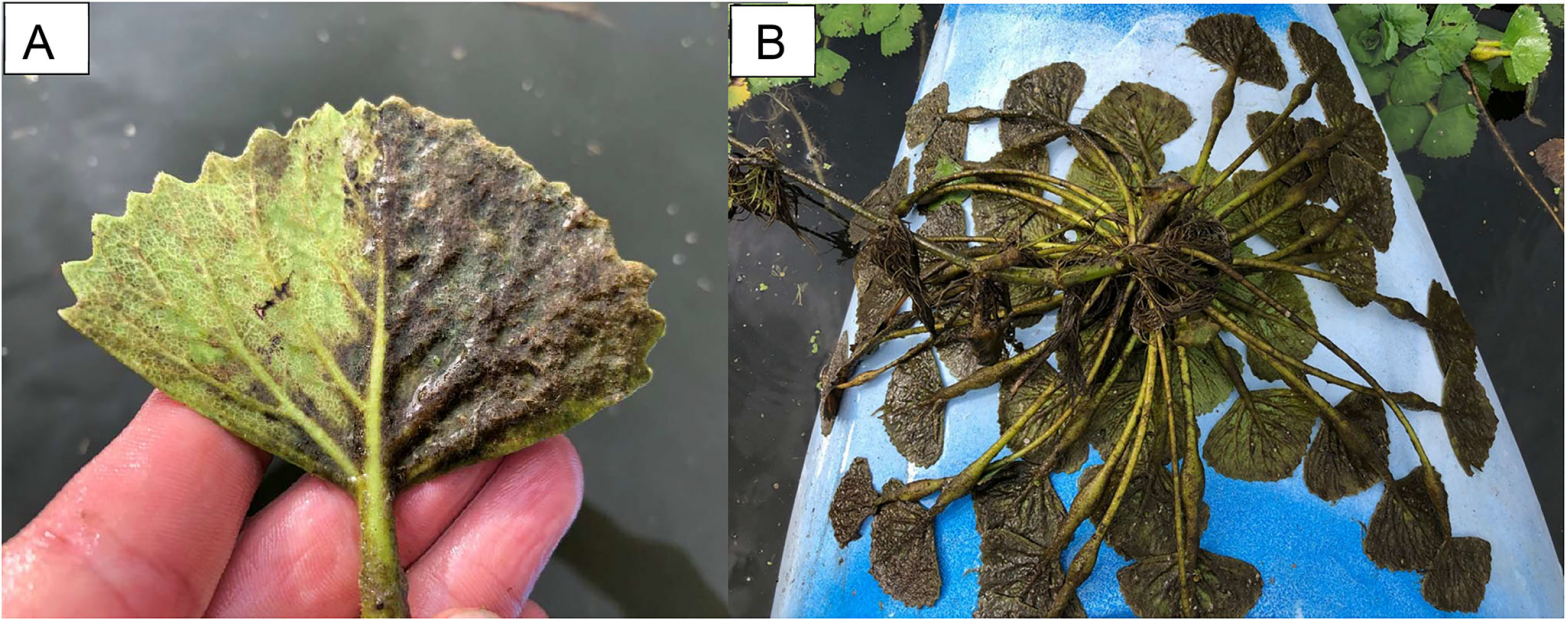
Biofilm on submerged plant parts of *T.natans*. On the left panel (A), the underside of a *T. natans* leaf, with biofilms scraped off the left but not the right hand side. On the right panel (B), the underside of an entire *T. natans* rosette. Note the difference in color between the dark submerged underside of the leaves, where biofilms are discernible, and the bright green adaxial surfaces where biofilms are not apparent.

Beyond denitrification, alternative pathways may contribute to the depletion of nitrate within *T. natans* beds. In addition to acquiring N from sediment pools, *T. natans* can directly assimilate nitrate from the water column and has been promoted as a phytoremediation species in its native range (Palm et al., 2024; Xu et al., 2020). Depletion of water‐column nitrate within *T. natans* beds may therefore reflect a combination of biological uptake, respiratory denitrification occurring in habitats not represented by our incubation design, and alternative microbially mediated pathways of nitrate transformation. For example, dissimilatory nitrate reduction to ammonium (DNRA) could retain nitrate‐derived N within the system as ammonium rather than remove it as N_2_ (Burgin & Hamilton, 2007). Distinguishing among these pathways is important because they have different implications for the fate of nitrogen. Plant uptake and DNRA retain nitrogen within the system, whereas denitrification represents permanent nitrogen removal. Future studies combining ^1^ ^5^N tracer approaches (Groffman et al., 2006) with measurements of denitrification in sediments, on plant surfaces, and within associated biofilms will be needed to quantify the relative importance of these pathways and determine the extent to which *T. natans* promotes permanent removal of nitrogen.

We frequently recorded DO concentrations <2 mg L^−1^, a lethal condition for many sensitive aquatic organisms (Caraco et al., 2006), and show that these conditions persist for many months of the annual growing season. Macrophyte beds are generally viewed as ecologically valuable because they provide refuge, increase habitat complexity, and support diverse aquatic communities (Burks et al., 2006; Cyr & Downing, 1988; Grenouillet & Pont, 2001; Meerhoff et al., 2007; Vadeboncoeur et al., 2011). However, these benefits assume that environmental conditions remain suitable for aquatic life. Although some invasive species may provide habitat benefits similar to native plants (Misteli et al., 2026; Tasker et al., 2022), high biomass and density can enable them to strongly control environmental conditions and homogenize habitats, potentially reducing their value for some native species. In contrast to these typical refuge effects, the persistent hypoxic conditions we document within *T. natans* beds suggest that these habitats may function as ecological traps or seasonal exclusion zones.

How extreme conditions in *T. natans* beds affect aquatic organisms at individual or population levels remains poorly understood but may be consequential. Mobile organisms, such as fish, may leave *T. natans* beds as hypoxic conditions become more severe over the growing season. However, in freshwater systems where much of the littoral zone is occupied by *T. natans*, alternatives may not exist, even for mobile organisms. Less mobile or sessile organisms, including freshwater mussels, benthic invertebrates, and other plants, may have fewer opportunities to escape persistent oxygen depletion within *T. natans* beds. Where suitable adjacent habitat is limited or low‐oxygen conditions extend beyond invaded beds, these habitats may become seasonally unsuitable (but see Kornijów et al., 2010). The ecological impacts of *T. natans* on the chemistry and food webs of freshwaters will therefore depend on the proportion of the area occupied and the hydrological characteristics of the affected system, with the greatest impacts expected where dense stands dominate shallow, poorly mixed waters.

In summary, we document strong ecosystem engineering effects of *T. natans* in nontidal systems, where dense plant beds consistently reduced DO and NO_3_^−^‐N during the middle of the growing season. However, the magnitude and spatial extent of these effects varied by hydrologic context, with impacts remaining localized in well‐mixed systems but extending downstream where dilution is limited. Although persistent hypoxia was accompanied by depletion of NO_3_^−^ ‐N, the fate of nitrogen remains unresolved. Management of *T. natans* may involve tradeoffs between restoring DO concentrations to benefit native biota and preserving potentially beneficial nitrogen‐removal functions. Understanding these tradeoffs will be important for predicting ecosystem responses to invasion and for designing effective management strategies.

## AUTHOR CONTRIBUTIONS

**Wade Simmons**: Conceptualization; data curation; formal analysis; funding acquisition; investigation; methodology; project administration; resources; software; validation; visualization; writing—original draft; writing—review and editing. **Bernd Blossey**: Conceptualization; funding acquisition; methodology; resources; supervision; writing—review and editing. **Michael Todd Walter**: Conceptualization; methodology; resources; supervision; writing—review and editing.

## CONFLICT OF INTEREST STATEMENT

The authors declare no conflicts of interest.

## DECLARATION OF GENERATIVE AI AND AI‐ASSISTED TECHNOLOGIES

During the preparation of this work, the authors used ChatGPT (OpenAI, GPT‐5.5) to assist with improving the clarity and readability of sections of this manuscript. After using this tool, the authors reviewed and edited all AI‐assisted content as needed, verified its accuracy, and take full responsibility for the content of the published article.

## Data Availability

Data will be made available upon reasonable request.
